# 2-(3-Hy­droxy­benzyl­amino)­acetic acid

**DOI:** 10.1107/S1600536811024226

**Published:** 2011-06-25

**Authors:** Li-Hua Zhi, Wei-Na Wu

**Affiliations:** a Department of Physics and Chemistry, Henan Polytechnic University, Jiaozuo 454000, People’s Republic of China

## Abstract

There are two independent 2-(3-hy­droxy­benzyl­amino)­acetic acid mol­ecules, C_9_H_11_NO_3_, in the asymmetric unit of the title compound. The dihedral angle between the benzene rings of the two independent mol­ecules is 58.12 (4)°. The crystal packing is stablized by inter­molecular O—H⋯O and N—H⋯O hydrogen bonds.

## Related literature

For the anti-tumor and artificial nuclease activity of copper complexes with substituted amino acid ligands, see: Jia *et al.* (2010 ▶).
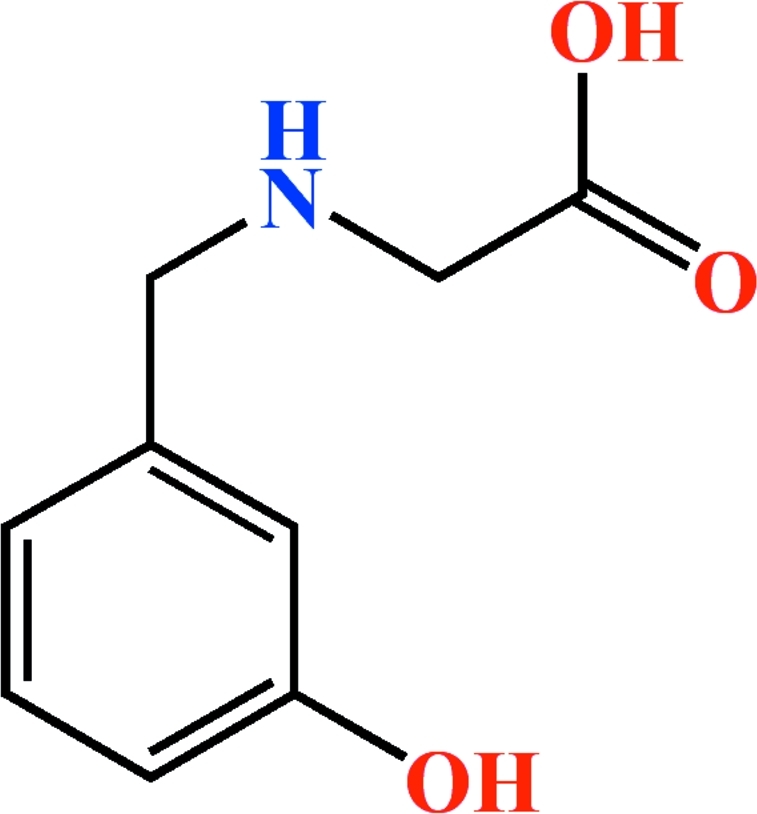


## Experimental

### 

#### Crystal data

C_9_H_11_NO_3_
*M* *_r_* = 181.19
Monoclinic, 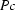

*a* = 11.9779 (3) Å
*b* = 8.0267 (2) Å
*c* = 9.3835 (2) Å
β = 101.391 (2)°
*V* = 884.39 (4) Å^3^
*Z* = 4
Mo *K*α radiation
μ = 0.10 mm^−1^
*T* = 296 K
0.23 × 0.16 × 0.12 mm

#### Data collection

Bruker APEXII CCD diffractometer
Absorption correction: multi-scan (*SADABS*; Bruker, 2007 ▶) *T*_min_ = 0.980, *T*_max_ = 0.988
7436 measured reflections
1999 independent reflections
1815 reflections with *I* > 2σ(*I*)
*R*_int_ = 0.022

#### Refinement

*R*[*F* ^2^ > 2σ(*F* ^2^)] = 0.043
*wR*(*F* ^2^) = 0.134
*S* = 1.07
1999 reflections
235 parameters
2 restraints
H-atom parameters constrained
Δρ_max_ = 0.40 e Å^−3^
Δρ_min_ = −0.32 e Å^−3^

### 

Data collection: *APEX2* (Bruker, 2007 ▶); cell refinement: *SAINT* (Bruker, 2007 ▶); data reduction: *SAINT*; program(s) used to solve structure: *SHELXS97* (Sheldrick, 2008 ▶); program(s) used to refine structure: *SHELXL97* (Sheldrick, 2008 ▶); molecular graphics: *SHELXTL* (Sheldrick, 2008 ▶); software used to prepare material for publication: *SHELXTL*.

## Supplementary Material

Crystal structure: contains datablock(s) I, global. DOI: 10.1107/S1600536811024226/vm2101sup1.cif

Structure factors: contains datablock(s) I. DOI: 10.1107/S1600536811024226/vm2101Isup2.hkl

Supplementary material file. DOI: 10.1107/S1600536811024226/vm2101Isup3.cml

Additional supplementary materials:  crystallographic information; 3D view; checkCIF report

## Figures and Tables

**Table 1:** Hydrogen-bond geometry (Å, °)

*D*—H⋯*A*	*D*—H	H⋯*A*	*D*⋯*A*	*D*—H⋯*A*
N1—H1*A*⋯O4	0.86	2.25	2.891 (3)	132
O3—H3*C*⋯O2^i^	0.82	1.84	2.639 (4)	166
N2—H2*C*⋯O2^ii^	0.86	2.28	2.910 (3)	130
O6—H6*A*⋯O4^iii^	0.82	1.85	2.646 (4)	165

Symmetry codes: (i) 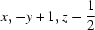; (ii) 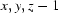; (iii) 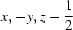.

**Fig. 1.**
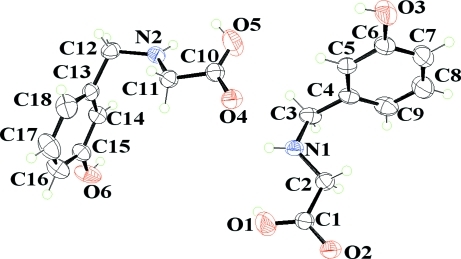
The title compound with the displacement ellipsoids shown at the 50% probability level.

**Fig. 2.**
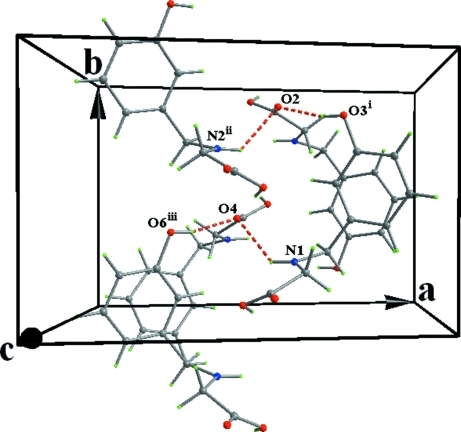
Part of the crystal packing for the title compound [hydrogen bonds shown as dashed lines, with symmetry codes: (i) x, -y + 1, z - 1/2; (ii) x, y, z - 1; (iii) x, -y, z - 1/2.].

## supplementary crystallographic information

### Comment

In recent years, substituted amino acid complexes have received extensive attention because of primarily their biological and pharmaceutical activities (Lei Jia *et al.*, 2010). As part of our studies of substituted amino acids, the title compound was synthesized and characterized by X-ray diffraction.

The asymmetric of the title compound, 2C_9_H_11_NO_3_, contains two independent 2-(3-hydroxybenzylamino)acetic acid molecules (Fig. 1). Each benzene ring is essentially planar [mean deviations of 0.0066 Å for ring C4—C9 and 0.0030 Å for ring C13—C18]. The torsion angles C12—N2 –C11—C10 and C2—N1—C3—C4 are -163.2 (2)° and -55.5 (3)°, respectively. The dihedral angle between the benzene rings in two independent amino acid molecules is 58.12 (4)°. In the crystal structure, intermolecular O—H···O and N—H···O hydrogen bonds are helpful to stabilize the packing (Table 1, Fig. 2).

### Experimental

To a clear solution of glycine (0.75 g, 10 mmol) and NaOH (0.40 g, 10 mmol) in a solvent mixture of water (10 mL) and methanol (20 mL), was added 3-hydroxy benzaldehyde (1.22 g, 10 mmol) and the resulting yellow solution was stirred for 3 h. After cooling to 273 K, a slight excess of NaBH_4_ (0.46 g, 12 mmol) was added. The yellow color slowly discharged after 20–30 min and the pH value was maintained 5–6 by addition of acetic acid. Colorless blocks of the title compound were obtained by slow evaporation of the reaction mixture.

### Refinement

We have merged Friedel-pair reflections before final refinement, as there is a light atom structure (heaviest element lighter than silicon, with Mo radiation). All H atoms were placed in calculated positions, with C—H = 0.93 and 0.97 Å, N—H = 0.86 Å and O—H = 0.82 Å, and were thereafter treated as riding, with *U*_iso_(H) values of 1.5*U*_eq_(O) for hydoxyl group and 1.2*U*_eq_(C,N) for others.

### Crystal data

**Table:**

C_9_H_11_NO_3_	*F*(000) = 384
*M**_r_* = 181.19	*D*_x_ = 1.361 Mg m^−^^3^
Monoclinic, *P**c*	Mo *K*α radiation, λ = 0.71073 Å
Hall symbol: P -2yc	Cell parameters from 3325 reflections
*a* = 11.9779 (3) Å	θ = 3.1–26.5°
*b* = 8.0267 (2) Å	µ = 0.10 mm^−^^1^
*c* = 9.3835 (2) Å	*T* = 296 K
β = 101.391 (2)°	Block, colorless
*V* = 884.39 (4) Å^3^	0.23 × 0.16 × 0.12 mm
*Z* = 4	

### Data collection

**Table:**

Bruker APEXII CCD diffractometer	1999 independent reflections
Radiation source: fine-focus sealed tube	1815 reflections with *I* > 2σ(*I*)
graphite	*R*_int_ = 0.022
φ and ω scans	θ_max_ = 27.4°, θ_min_ = 1.7°
Absorption correction: multi-scan (*SADABS*; Bruker, 2007)	*h* = −15→14
*T*_min_ = 0.980, *T*_max_ = 0.988	*k* = −10→10
7436 measured reflections	*l* = −12→12

### Refinement

**Table:**

Refinement on *F*^2^	Primary atom site location: structure-invariant direct methods
Least-squares matrix: full	Secondary atom site location: difference Fourier map
*R*[*F*^2^ > 2σ(*F*^2^)] = 0.043	Hydrogen site location: inferred from neighbouring sites
*wR*(*F*^2^) = 0.134	H-atom parameters constrained
*S* = 1.07	*w* = 1/[σ^2^(*F*_o_^2^) + (0.0892*P*)^2^ + 0.1326*P*] where *P* = (*F*_o_^2^ + 2*F*_c_^2^)/3
1999 reflections	(Δ/σ)_max_ = 0.007
235 parameters	Δρ_max_ = 0.40 e Å^−^^3^
2 restraints	Δρ_min_ = −0.32 e Å^−^^3^

### Special details

**Table:**

Geometry. All e.s.d.'s (except the e.s.d. in the dihedral angle between two l.s. planes) are estimated using the full covariance matrix. The cell e.s.d.'s are taken into account individually in the estimation of e.s.d.'s in distances, angles and torsion angles; correlations between e.s.d.'s in cell parameters are only used when they are defined by crystal symmetry. An approximate (isotropic) treatment of cell e.s.d.'s is used for estimating e.s.d.'s involving l.s. planes.
Refinement. Refinement of *F*^2^ against ALL reflections. The weighted *R*-factor *wR* and goodness of fit *S* are based on *F*^2^, conventional *R*-factors *R* are based on *F*, with *F* set to zero for negative *F*^2^. The threshold expression of *F*^2^ > σ(*F*^2^) is used only for calculating *R*-factors(gt) *etc*. and is not relevant to the choice of reflections for refinement. *R*-factors based on *F*^2^ are statistically about twice as large as those based on *F*, and *R*- factors based on ALL data will be even larger.

### Fractional atomic coordinates and isotropic or equivalent isotropic displacement parameters (Å^2^)

**Table:**

	*x*	*y*	*z*	*U*_iso_*/*U*_eq_	
O2	0.55759 (19)	0.0934 (3)	0.6066 (2)	0.0384 (5)	
N1	0.6095 (2)	0.2130 (3)	0.2543 (3)	0.0336 (5)	
H1A	0.5405	0.2099	0.2063	0.040*	
C1	0.5447 (3)	0.0976 (4)	0.4692 (3)	0.0359 (6)	
C4	0.7774 (3)	0.3969 (3)	0.2604 (3)	0.0345 (6)	
O3	0.7554 (3)	0.8513 (3)	0.2813 (4)	0.0615 (8)	
H3C	0.6936	0.8514	0.2251	0.092*	
C9	0.8794 (3)	0.3737 (4)	0.3539 (5)	0.0491 (9)	
H9	0.9079	0.2665	0.3728	0.059*	
O1	0.4643 (3)	0.0409 (5)	0.3829 (3)	0.0810 (12)	
H1B	0.4864	0.0058	0.3112	0.121*	
C5	0.7337 (3)	0.5573 (3)	0.2338 (3)	0.0354 (6)	
H5	0.6644	0.5739	0.1705	0.042*	
C3	0.7094 (3)	0.2534 (4)	0.1859 (3)	0.0396 (7)	
H3A	0.6824	0.2806	0.0842	0.048*	
H3B	0.7581	0.1561	0.1906	0.048*	
C6	0.7942 (3)	0.6920 (4)	0.3025 (4)	0.0420 (7)	
C2	0.6411 (3)	0.1792 (4)	0.4121 (3)	0.0353 (6)	
H2A	0.6614	0.2830	0.4636	0.042*	
H2B	0.7073	0.1069	0.4309	0.042*	
C7	0.8982 (4)	0.6685 (5)	0.3941 (5)	0.0542 (9)	
H7	0.9395	0.7595	0.4375	0.065*	
C8	0.9407 (3)	0.5093 (5)	0.4211 (5)	0.0598 (11)	
H8	1.0102	0.4926	0.4841	0.072*	
O4	0.4311 (2)	0.4024 (3)	0.0722 (2)	0.0387 (5)	
N2	0.3782 (2)	0.2918 (3)	−0.3084 (3)	0.0320 (5)	
H2C	0.4470	0.2960	−0.3224	0.038*	
C10	0.4428 (3)	0.4038 (4)	−0.0589 (3)	0.0353 (6)	
O6	0.2384 (3)	−0.3410 (3)	−0.3402 (4)	0.0595 (8)	
H6A	0.2981	−0.3414	−0.3704	0.089*	
C13	0.2093 (3)	0.1109 (3)	−0.3881 (3)	0.0356 (6)	
O5	0.5208 (3)	0.4690 (5)	−0.1059 (3)	0.0742 (10)	
H5A	0.4970	0.5018	−0.1891	0.111*	
C14	0.2554 (3)	−0.0481 (4)	−0.3873 (3)	0.0355 (6)	
H14	0.3250	−0.0643	−0.4151	0.043*	
C11	0.3485 (3)	0.3195 (4)	−0.1654 (3)	0.0360 (6)	
H11A	0.3313	0.2132	−0.1255	0.043*	
H11B	0.2804	0.3878	−0.1777	0.043*	
C18	0.1069 (3)	0.1349 (4)	−0.3470 (5)	0.0503 (8)	
H18	0.0764	0.2415	−0.3476	0.060*	
C12	0.2773 (3)	0.2555 (4)	−0.4279 (3)	0.0400 (7)	
H12A	0.3035	0.2299	−0.5169	0.048*	
H12B	0.2290	0.3534	−0.4454	0.048*	
C15	0.1966 (3)	−0.1827 (4)	−0.3447 (4)	0.0392 (7)	
C16	0.0943 (3)	−0.1573 (5)	−0.3026 (5)	0.0509 (9)	
H16	0.0558	−0.2473	−0.2726	0.061*	
C17	0.0483 (3)	−0.0004 (5)	−0.3043 (6)	0.0575 (10)	
H17	−0.0215	0.0155	−0.2772	0.069*	

### Atomic displacement parameters (Å^2^)

**Table:**

	*U*^11^	*U*^22^	*U*^33^	*U*^12^	*U*^13^	*U*^23^
O2	0.0484 (12)	0.0335 (11)	0.0343 (11)	−0.0017 (9)	0.0108 (9)	0.0037 (8)
N1	0.0403 (13)	0.0270 (11)	0.0340 (12)	−0.0019 (10)	0.0086 (10)	0.0040 (10)
C1	0.0409 (15)	0.0337 (14)	0.0340 (15)	−0.0043 (11)	0.0098 (12)	0.0009 (11)
C4	0.0388 (14)	0.0299 (13)	0.0394 (14)	−0.0002 (11)	0.0191 (12)	0.0034 (11)
O3	0.0655 (16)	0.0237 (10)	0.083 (2)	0.0011 (11)	−0.0151 (14)	−0.0043 (11)
C9	0.0442 (18)	0.0337 (15)	0.071 (2)	0.0074 (13)	0.0154 (17)	0.0101 (15)
O1	0.0679 (18)	0.133 (3)	0.0427 (14)	−0.054 (2)	0.0118 (13)	−0.0031 (17)
C5	0.0395 (15)	0.0279 (14)	0.0384 (15)	0.0006 (11)	0.0070 (12)	0.0048 (12)
C3	0.0541 (18)	0.0300 (14)	0.0400 (17)	−0.0011 (12)	0.0220 (14)	−0.0007 (11)
C6	0.0466 (17)	0.0289 (14)	0.0492 (18)	−0.0003 (12)	0.0064 (14)	0.0031 (13)
C2	0.0382 (14)	0.0379 (15)	0.0320 (13)	−0.0036 (12)	0.0120 (11)	−0.0017 (11)
C7	0.055 (2)	0.0395 (17)	0.063 (2)	−0.0074 (15)	−0.0017 (17)	0.0021 (16)
C8	0.0396 (17)	0.051 (2)	0.081 (3)	0.0002 (15)	−0.0062 (17)	0.0135 (19)
O4	0.0513 (13)	0.0331 (11)	0.0325 (10)	−0.0013 (9)	0.0099 (9)	−0.0041 (8)
N2	0.0355 (12)	0.0259 (10)	0.0353 (12)	−0.0016 (9)	0.0092 (10)	−0.0023 (9)
C10	0.0418 (15)	0.0323 (13)	0.0319 (14)	−0.0026 (11)	0.0077 (12)	−0.0033 (11)
O6	0.0686 (17)	0.0265 (10)	0.093 (2)	0.0015 (11)	0.0384 (16)	0.0071 (12)
C13	0.0411 (15)	0.0299 (14)	0.0325 (14)	−0.0031 (11)	−0.0009 (11)	−0.0027 (11)
O5	0.0715 (18)	0.109 (3)	0.0444 (15)	−0.0508 (19)	0.0164 (13)	−0.0151 (16)
C14	0.0383 (14)	0.0318 (14)	0.0376 (14)	0.0005 (11)	0.0106 (12)	−0.0050 (12)
C11	0.0416 (15)	0.0364 (14)	0.0301 (13)	−0.0045 (12)	0.0075 (11)	−0.0013 (11)
C18	0.0451 (18)	0.0396 (17)	0.065 (2)	0.0081 (15)	0.0079 (16)	−0.0067 (16)
C12	0.0567 (19)	0.0284 (13)	0.0324 (15)	−0.0041 (13)	0.0028 (14)	0.0005 (11)
C15	0.0428 (16)	0.0300 (14)	0.0451 (17)	−0.0035 (12)	0.0095 (13)	−0.0038 (12)
C16	0.053 (2)	0.0401 (17)	0.063 (2)	−0.0113 (15)	0.0201 (17)	−0.0053 (16)
C17	0.0388 (17)	0.052 (2)	0.085 (3)	−0.0011 (15)	0.0225 (17)	−0.0100 (19)

### Geometric parameters (Å, °)

**Table:**

O2—C1	1.268 (4)	O4—C10	1.266 (4)
N1—C2	1.479 (4)	N2—C11	1.472 (4)
N1—C3	1.501 (4)	N2—C12	1.507 (4)
N1—H1A	0.8600	N2—H2C	0.8600
C1—O1	1.217 (4)	C10—O5	1.226 (4)
C1—C2	1.514 (4)	C10—C11	1.512 (4)
C4—C9	1.369 (5)	O6—C15	1.362 (4)
C4—C5	1.394 (4)	O6—H6A	0.8200
C4—C3	1.501 (4)	C13—C18	1.370 (5)
O3—C6	1.362 (4)	C13—C14	1.390 (4)
O3—H3C	0.8200	C13—C12	1.507 (4)
C9—C8	1.392 (6)	O5—H5A	0.8200
C9—H9	0.9300	C14—C15	1.391 (4)
O1—H1B	0.8200	C14—H14	0.9300
C5—C6	1.387 (4)	C11—H11A	0.9700
C5—H5	0.9300	C11—H11B	0.9700
C3—H3A	0.9700	C18—C17	1.394 (6)
C3—H3B	0.9700	C18—H18	0.9300
C6—C7	1.380 (5)	C12—H12A	0.9700
C2—H2A	0.9700	C12—H12B	0.9700
C2—H2B	0.9700	C15—C16	1.376 (5)
C7—C8	1.381 (6)	C16—C17	1.373 (6)
C7—H7	0.9300	C16—H16	0.9300
C8—H8	0.9300	C17—H17	0.9300
			
C2—N1—C3	113.6 (2)	C11—N2—C12	113.9 (2)
C2—N1—H1A	123.2	C11—N2—H2C	123.1
C3—N1—H1A	123.2	C12—N2—H2C	123.1
O1—C1—O2	126.0 (3)	O5—C10—O4	126.3 (3)
O1—C1—C2	119.0 (3)	O5—C10—C11	118.5 (3)
O2—C1—C2	115.0 (3)	O4—C10—C11	115.2 (3)
C9—C4—C5	119.8 (3)	C15—O6—H6A	109.5
C9—C4—C3	121.8 (3)	C18—C13—C14	120.3 (3)
C5—C4—C3	118.4 (3)	C18—C13—C12	121.3 (3)
C6—O3—H3C	109.5	C14—C13—C12	118.4 (3)
C4—C9—C8	120.6 (3)	C10—O5—H5A	109.5
C4—C9—H9	119.7	C15—C14—C13	119.4 (3)
C8—C9—H9	119.7	C15—C14—H14	120.3
C1—O1—H1B	109.5	C13—C14—H14	120.3
C6—C5—C4	119.6 (3)	N2—C11—C10	112.7 (2)
C6—C5—H5	120.2	N2—C11—H11A	109.1
C4—C5—H5	120.2	C10—C11—H11A	109.1
N1—C3—C4	111.8 (2)	N2—C11—H11B	109.1
N1—C3—H3A	109.3	C10—C11—H11B	109.1
C4—C3—H3A	109.3	H11A—C11—H11B	107.8
N1—C3—H3B	109.3	C13—C18—C17	120.0 (3)
C4—C3—H3B	109.3	C13—C18—H18	120.0
H3A—C3—H3B	107.9	C17—C18—H18	120.0
O3—C6—C7	117.3 (3)	N2—C12—C13	111.0 (2)
O3—C6—C5	122.2 (3)	N2—C12—H12A	109.4
C7—C6—C5	120.5 (3)	C13—C12—H12A	109.4
N1—C2—C1	111.8 (2)	N2—C12—H12B	109.4
N1—C2—H2A	109.3	C13—C12—H12B	109.4
C1—C2—H2A	109.3	H12A—C12—H12B	108.0
N1—C2—H2B	109.3	O6—C15—C16	118.2 (3)
C1—C2—H2B	109.3	O6—C15—C14	122.0 (3)
H2A—C2—H2B	107.9	C16—C15—C14	119.8 (3)
C8—C7—C6	119.7 (3)	C17—C16—C15	120.7 (3)
C8—C7—H7	120.1	C17—C16—H16	119.6
C6—C7—H7	120.1	C15—C16—H16	119.6
C7—C8—C9	119.8 (3)	C16—C17—C18	119.7 (3)
C7—C8—H8	120.1	C16—C17—H17	120.2
C9—C8—H8	120.1	C18—C17—H17	120.2
			
C5—C4—C9—C8	−1.0 (5)	C18—C13—C14—C15	0.1 (5)
C3—C4—C9—C8	179.8 (4)	C12—C13—C14—C15	177.2 (3)
C9—C4—C5—C6	0.1 (5)	C12—N2—C11—C10	−163.2 (2)
C3—C4—C5—C6	179.3 (3)	O5—C10—C11—N2	14.3 (5)
C2—N1—C3—C4	−55.5 (3)	O4—C10—C11—N2	−167.2 (3)
C9—C4—C3—N1	103.7 (4)	C14—C13—C18—C17	−0.1 (5)
C5—C4—C3—N1	−75.5 (3)	C12—C13—C18—C17	−177.1 (4)
C4—C5—C6—O3	−179.7 (3)	C11—N2—C12—C13	−54.6 (3)
C4—C5—C6—C7	1.4 (5)	C18—C13—C12—N2	105.2 (3)
C3—N1—C2—C1	−165.6 (2)	C14—C13—C12—N2	−71.9 (4)
O1—C1—C2—N1	13.6 (5)	C13—C14—C15—O6	−179.2 (3)
O2—C1—C2—N1	−167.4 (3)	C13—C14—C15—C16	−0.6 (5)
O3—C6—C7—C8	179.0 (4)	O6—C15—C16—C17	179.7 (4)
C5—C6—C7—C8	−2.0 (6)	C14—C15—C16—C17	1.1 (6)
C6—C7—C8—C9	1.1 (7)	C15—C16—C17—C18	−1.0 (6)
C4—C9—C8—C7	0.4 (7)	C13—C18—C17—C16	0.5 (6)

### Hydrogen-bond geometry (Å, °)

**Table:**

*D*—H···*A*	*D*—H	H···*A*	*D*···*A*	*D*—H···*A*
N1—H1A···O4	0.86	2.25	2.891 (3)	132
O3—H3C···O2^i^	0.82	1.84	2.639 (4)	166
N2—H2C···O2^ii^	0.86	2.28	2.910 (3)	130
O6—H6A···O4^iii^	0.82	1.85	2.646 (4)	165

Symmetry codes: (i) *x*, −*y*+1, *z*−1/2; (ii) *x*, *y*, *z*−1; (iii) *x*, −*y*, *z*−1/2.
